# Acute respiratory symptoms and evacuation-related behavior after exposure to chlorine gas leakage

**DOI:** 10.1186/s40557-016-0115-2

**Published:** 2016-07-04

**Authors:** Sung-Woo Han, Won-Jun Choi, Min-Kee Yi, Seng-Ho Song, Dong-Hoon Lee, Sang-Hwan Han

**Affiliations:** Department of Occupational & Environmental Medicine, Gachon University Gil Medical Center, Incheon, Korea

**Keywords:** Chlorine, Gas leakage, Chemical hazard, Evacuation behavior

## Abstract

**Background:**

A study was performed on the accidental chlorine gas leakage that occurred in a factory of printed circuit boards manufactured without chlorine. Health examination was performed for all 52 workers suspected of exposure to chlorine gas, and their evacuation-related behaviors were observed in addition to analyzing the factors that affected the duration of their acute respiratory symptoms.

**Methods:**

Behavioral characteristics during the incidence of the accidental chlorine gas leakage, the estimated time of exposure, and the duration of subjective acute respiratory symptoms were investigated. In addition, clinical examination, chest radiography, and dental erosion test were performed. As variables that affected the duration of respiratory symptoms, dose group, body weight, age, sex, smoking, work period, and wearing a protective gear were included and analyzed by using the Cox proportional hazard model.

**Results:**

Of 47 workers exposed to chlorine gas, 36 (77 %) developed more than one subjective symptom. The duration of the subjective symptoms according to exposure level significantly differed, with a median of 1 day (range, 0–5 days) in the low-exposure group and 2 days (range, 0–25 days) in the high-exposure group. Among the variables that affected the duration of the acute respiratory symptoms, which were analyzed by using the Cox proportional hazard model, only exposure level was significant (hazard ratio 2.087, 95 % CI = 1.119, 3.890). Regarding the evacuation-related behaviors, 22 workers (47 %) voluntarily evacuated to a safety zone immediately after recognizing the accidental exposure, but 25 workers (43 %) delayed evacuation until the start of mandatory evacuation (min 5, max 25 min).

**Conclusions:**

The duration of the subjective acute respiratory symptoms significantly differed between the low- and high-exposure groups. Among the 27 workers in the high-exposure group, 17 misjudged the toxicity after being aware of the gas leakage, which is a relatively high number.

## Background

Chlorine is a yellow-green toxic gas with a foul odor that is 2.5 times denser than air. It exists as a diatomic molecule under standard conditions and is broadly used as an oxidizer, bleach, and disinfectant [1, 2]. Chlorine gas is water soluble and irritates the upper airway and mucous membranes in acute exposure. Irritation of the upper airway can occur at a concentration of 1 ppm, which can cause skin pigmentation and dermatitis, hemoptysis, dyspnea, and chest pain at high-concentration and prolonged exposure [3, 4]. Although several institutions have reported the health risk of exposure at certain chlorine gas dosages, the Acute Exposure Guideline Level, which was developed by the United States Environmental Protection Agency, classifies the risk of exposure duration and atmospheric concentration of chlorine gas in inhalation exposure and is being broadly used. Class 1 is defined as tolerable dose when exposure is at 0.5 ppm for 10 min. Class 2 is defined as dose that can cause irreversible damage when exposure is at 2.8 ppm for more than 10 min. Class 3 is defined as dose that can be fatal when exposure is at 50 ppm for more than 10 min [5, 6].

This study was performed on the basis of data obtained from temporary health examinations of the victims of an accidental chlorine gas leakage at a printed circuit board factory in August 2014. The leakage was characterized by chlorine gas produced in the factory, which did not use chlorine as a raw material. The causes of the accident were found to be the large amount of sodium chlorate flowing into the waste storage tank as result of a mistake made by a worker and the piping design error. Chlorine gas was produced as a product of a reaction of sodium chlorate with the copper chloride in the waste, both of which were leaked into the inside of the factory, as the factory had no independent ventilation facility for its waste storage tank. When a workplace is at risk of an occupational disaster, temporary health examination is performed for all workers according to the Korean Industrial Safety and Health Act in order to evaluate the effects of the risk factors on their health and to suggest whether the workplace environment is still conducive for the workers [7].

Performing an appropriate evacuation behavior can reduce exposure time when accidental leakage of hazardous materials occurs and is the most significant factor in influencing health outcomes in this situation [8, 9]. We investigated the time taken for leakage of hazardous materials to be recognized and the methods used in this process. We also assessed whether the evacuation time and route were appropriate and estimated the expected exposure time to the chlorine gas. In addition, acute effects on health due to chlorine gas exposure were evaluated, and factors affecting the period for which subjective acute respiratory symptoms persisted were analyzed. For this, we investigated and analyzed the evacuation-related behaviors of 52 workers who were considered to have been exposed during the accidental chlorine gas leakage, as well as performing physical examinations. Although the effect of accidental chlorine gas leakage on health has been reported, reports on evaluations of all victims who are suspected of being exposed to chlorine gas, as in this study, are rare.

## Methods

### Temporary health examination

This study used data from medical records in the emergency department and results of the temporary health examinations of the workers exposed to chlorine gas produced by a small PCB factory located in Incheon, South Korea, in August 2014. The factory was about 400 square meters in total building floor area and consisted of 80 workers. The accidental chlorine gas leakage occurred at around 7:00 am, and 52 people were inside the factory at the time of the incident. Temporary health examinations were performed for all the 52 workers who were working on the day of the accident and were suspected of being exposed to chlorine gas, including 25 people who received emergency treatment at the time of the accident. After excluding the 5 workers who were determined to have had no significant chlorine exposure based on the evaluation questionnaire about exposure time, data from the medical records of 47 people were used for statistical analysis.

Temporary health examination was performed after Walk-through survey of the place of the incident, for 3 days, from the seventh day of the accidental leakage. All the subjects answered a self-administered survey questionnaire and received clinical examinations and tests. Physical examination, chest radiography, and dental erosion test were performed. When the basic examination revealed no abnormal findings, chest computed tomography was additionally performed. The test items were based on the chlorine gas items listed under each health examination method corresponding to each hazardous agent in the practical guide for workers’ health examination published by the Occupational Safety and Health Research Institute and were determined by consulting other references related to the health effects of chlorine gas inhalation [1, 2, 7]. Clinical examination was conducted by a physician, and chest radiography readings were performed by a radiologist. The dental erosion tests were performed by a dentist. When the subjects had received emergency treatment prior to the temporary health examination, their medical records were reflected on the evaluation for health effects.

Chlorine gas exposure time was defined as the time from the start of chlorine gas exposure to the time of evacuation to the area outside the building that was designated as a safety zone. The time and method of recognition of the gas leakage, the location of the leakage, and the beginning and end time of the evacuation were investigated through interviews. Individual timelines were created. Regarding the evacuation behaviors, the recognition process of the accidental gas leakage; the decision-making process corresponding to each evacuation behavior; the evacuation routes; the evacuation methods; the reason for delayed evacuation, if any; and whether protective gear was worn were investigated. The lasting period of acute respiratory symptoms was defined as the number of days that at least one subjective symptom, such as a sore throat, coughing, sputum, wheezing, or other respiratory symptoms, lasted. The subjects who were exposed to chlorine gas for > and <10 min were classified as the high- and low-exposure groups, respectively. This study was approved by the Institutional Review Board of the Gacheon University Gil Hospital (GBIRB 2015-165).

### Statistical analysis

The *t*-test or Mann-Whitney *U* test was performed for continuous variables; and the chi-square test, for categorical variables. As variables that affected the duration of respiratory symptoms, dose group, body weight, age, sex, smoking, work period, and wearing a protective gear were included and analyzed by using the Cox proportional hazard model. The log negative log survival plot demonstrated the proportional hypothesis of the Cox proportional hazard model to verify that the two groups were parallel. Null hypothesis was declined when the significant probability was <0.5 in the two-tailed test.

## Results

### Acute health effects of chlorine gas

Of the 52 subjects, 5 were assessed as having had no significant chlorine gas exposure and 47 were classified into the low- (<10 min, *n* = 20) and high-exposure groups (>10 min, *n* = 27) (Table 1). For the variables “estimated exposure time” and “duration of subjective acute respiratory symptoms”, normality could not be hypothesized in the Shapiro-Wilk test (*p* < 0.000). For the anthropological characteristics of the subjects, the mean age in the high-exposure group was 45.0 years, which was significantly higher than the mean of 37.9 years in the low-exposure group. However, no significant differences in sex, work experience at the current workplace, and smoking history were found. The number of subjects with a history of respiratory diseases was too small sample to be appropriate for statistical management. The chlorine exposure time was estimated to range from 1 to 30 min. The median exposure time in the low-exposure group was 2 min (range, 1–8 min), and that in the high-exposure group was 10 min (range, 10–30 min). The duration of the subjective acute respiratory symptoms was 1 day (range, 0–5 days) in the low-exposure group and 2 days (range, 0–25 days) in the high-exposure group.

**Table 1:** Characteristics of the study subjects

Variables	Total (*n* = 47)		Low exposure (*n* = 20)		High exposure (*n* = 27)	
	n	%	n	%	n	%
Age (year)						
Mean ± SD*	41.70 ± 9.27		37.90 ± 9.89		45.00 ± 8.19	
Tenure (year)						
Mean ± SD	2.55 ± 1.80		2.45 ± 1.96		2.63 ± 1.71	
Exposure time (minute)						
Median (range)**	10 (1–30)		2 (1–8)		10 (10–30)	
Lasting respiratory symptoms (day)						
Median (range)**	1 (0–25)		1 (0–5)		2 (0–25)	
Sex						
Male	39	83.0	17	85.0	22	81.5
Female	8	17.0	3	15.0	5	18.5
Smoking status						
Never	20	42.6	10	50.0	10	37.0
Current	22	46.8	10	50.0	12	44.4
Former	5	10.6	0	0.0	5	18.5
Preexisting disease						
None	44	93.6	19	95.0	25	92.6
Asthma or COPD	0	0.0	0	0.0	0	0.0
Hypertension	2	4.3	0	0.	2	7.4
Dermatologic disease	1	2.1	1	5.0	0	0.0

*p value <0.05 by *t*-test

**p value <0.05 by Mann-Whitney *U* test

The 36 subjects exposed to chlorine gas reported more than one subjective acute respiratory symptom (Table 2). The most prevalent symptom was cough (28 cases), followed by sore throat (27 cases) and phlegm (17 cases). The prevalence of upper airway symptoms was high. Per exposure group, 12 of the 20 subjects in the low-exposure group complained of more than one respiratory symptom, including coughing in 11 subjects, sore throat in 7, and dizziness in 6. Among the 27 subjects in the high-exposure group, 24 complained of more than one respiratory symptom, including sore throat in 20 subjects, coughing in 17, and phlegm in 15. Cardiovascular, ocular irritation, and dermatological symptoms were not statistically significant factors, but showed higher prevalence rates in the high-exposure group. In the physical examination, one subject had coarse breath sounds on chest auscultation. In the chest radiography, one patient had local density on the left lower lobe of the lung was observed, and another patient had suspected pulmonary edema. None of the subjects showed abnormal findings in the dental erosion test.

**Table 2:** Reported Symptoms after chlorine exposure

Variables	Total (*n* = 47)		Low exposure (*n* = 20)		High exposure (*n* = 27)	
	n	%	n	%	n	%
One or more respiratory symptoms*	36	76.6	12	60.0	24	88.9
Throat pain*	27	57.4	7	35.0	20	74.1
Wheezing	15	31.9	3	15.0	12	44.4
Cough	28	59.6	11	55.0	17	63.0
Sputum	17	36.2	4	20.0	13	48.1
Cardiovascular						
Chest discomfort*	14	29.8	3	15.0	11	40.7
Palpitation	11	23.4	3	15.0	8	29.6
Ophthalmologic						
Sore eyes	10	21.3	2	10.0	8	29.6
Dermatologic						
Skin trouble	3	6.4	0	0.0	3	11.1
General symptom						
Agitation	2	4.3	1	5.0	1	3.7
Dizziness	16	34.0	5	25.0	11	40.7
Headache	9	19.1	3	15.0	6	22.2

*p < 0.05 by Chi-square test

In the analysis of the factors that affected the duration of acute respiratory symptoms by using the Cox proportional hazard model, only exposure group significantly affected the duration of the subjective acute respiratory symptoms, with a hazard ration of 2.087 (95 % CI = 1.119, 3.890). Age, sex, body weight, work period, and smoking history had no significant effects.

### Evacuation-related behaviors

The process by which hazardous gas leakage was recognized was investigated (Table 3). Three subjects (6 %) responded that they knew about the incident of the accidental leakage through the evacuation orders. The rest of the 44 subjects (94 %) responded that they directly recognized the incidence of accidental gas leakage by seeing, smelling, and experiencing a dermatological irritation. The 3 of the 44 subjects who directly recognized the accidental gas leakage responded that they thought the gas was chlorine gas, and 20 responded that they thought the gas was not identifiable but might be hazardous. The rest of the 21 subjects responded that they thought the gas would be harmless. The 3 subjects who recognized the accidental gas leakage through the evacuation orders started evacuation immediately after recognizing the accident. However, only 19 of the 44 who directly recognized the gas leakage immediately started evacuation, and the remaining 25 subjects did not evacuate but displayed other behaviors.

**Table 3:** Evacuation-related behavior after exposure to chlorine

Variables	Total (*n* = 47)		Low exposure (*n* = 20)		High exposure (*n* = 27)	
	n	%	n	%	n	%
Recognize gas leakage						
Direct^a^	44	93.6	17	85.0	27	100.0
Indirect^b^	3	6.4	3	15.0	0	0
Start evacuation*						
Evacuate immediately to the correct path^c^	16	34.0	16	80.0	0	0
Evacuate immediately to the incorrect path^d^	6	12.8	0	0	6	22.2
Evacuation is delayed	25	53.2	4	20.0	21	77.8

*p < 0.05 by Chi-square test

^a^Recognized gas leakage directly (visual, olfactory or skin sensation)

^b^Recognized gas leakage by receive evacuation instruction

^c^Evacuated to the emergency exit

^d^Evacuated to the main stairway adjacent to the chlorine gas source

In the investigation of the reasons of the 25 subjects who did not start evacuation immediately after recognizing the gas leakage, 21 responded that beginning evacuation was delayed because they continued working based on their judgment that the leaked gas might be harmless (Table 4). Although 3 subjects knew that it was hazardous gas, they delayed evacuation because they had to help other workers evacuation. One subject responded that evacuation was delayed because the subject had to identify the origin of the leakage and deal with the aftermath of the accident. The median start time of the delayed evacuation for the 25 subjects was 10 min (range, 5–25 min) after the onset of exposure.

**Table 4:** The reasons for the delay of start evacuation

Variables	Total (*n* = 25)	
	n	%
Didn’t know it was toxic gas	21	84.0
Knew a toxic gas, but help others evacuation	3	12.0
Try to check the gas leakage site	1	4.0

According to the estimated exposure time to chlorine gas, 21 subjects (78 %) in the high-exposure group had prolonged exposure due to delayed evacuation after recognition of the accidental gas leakage (Table 3). Six subjects (22 %) in the high-exposure group started evacuation immediately after recognizing the gas leakage but were exposed to a high dose, as it took >10 min to complete the evacuation to a safety zone. For whether a protective gear was worn during evacuation, 3 subjects responded that they evacuated with a protective gear on, but it was found that they were wearing dust-proof masks based on the field investigation.

## Discussion

Exposure time to chlorine gas differed according to an individual’s evacuation-related behaviors, and led to differences in the lasting period of subjective acute respiratory symptoms. The incidence rate of more than one acute respiratory symptom was higher and the duration of acute respiratory symptoms was significantly longer in the high-exposure group than in the low-exposure group. In the analysis of the effects of confounders, body weight, height, age, sex, smoking history, and work period, in addition to exposure dose, were found to have no significant effects the duration of acute respiratory symptoms. Results from the present study are similar to those from previous studies that have investigated acute health effects in accidental chlorine gas leakage [10–15].

The prevalence rate of more than one acute respiratory symptom was high in the high-exposure group, as well as the prevalence rates of chest discomfort and the cardiovascular symptoms. This result shows the characteristic of the chlorine gas that caused irritation of the upper airway during the early phase of exposure, which further affected the circulatory and nervous systems as the exposure time was prolonged. This study selected duration of subjective acute respiratory symptoms as a variable to assess the effects of chlorine gas. In this study, the duration of the subjective acute respiratory symptoms tended to be prolonged compared with physical findings by a physician or objective test results. Although the interview was performed by a physician, it recorded subjective complaints of symptoms; thus, recall bias was possible. In addition, symptoms of light coughing or phlegm were most frequently reported, as the exposure was to a hazardous agent. Thus, the duration of the subjective respiratory symptoms might have been more prolonged [2, 16, 17].

Evacuation-related behaviors are the most significant factors determining health outcomes in victims of disasters inside buildings. Studies of these behaviors have been reported by various researchers. Significant determinants of exposure time are whether people have made a quick decision about rapid evacuation in the early stages by recognizing the disaster, and whether they have chosen a proper evacuation route [8, 18]. In this case, the factory where the accident occurred was a modern four-floor building established in 2013 on a plot of land of about 400 m^2^. Based on the field investigation, it did not take more than 5 min to evacuate through main or alternative exits from anywhere in the building. It is worth paying attention to the reasons that the expected exposure time to chlorine gas varied from 2 min to 30 min. Based on our investigation, delays in pre-movement time and travel time affected 25 and 6 people, respectively. Two reasons for these results are as follows:

Firstly, only the process manager and a few workers were aware that chlorine gas could be produced as a byproduct of a chemical reaction used in the copper etching process. Most of the workers did not know that chlorine gas could be produced as a byproduct during the manufacturing process. The 17 subjects in the high-exposure group who recognized the chlorine gas leakage but could not determine whether it was a hazardous agent and thus delayed their time to begin evacuation, consequently prolonging their exposure time. The accident occurred 1 year since the start of factory operation. Although the company responsible for the accident had posted material safety data sheets on the hazardous materials used in the factory according to the Korean Industrial Safety and Health Act and periodic education was being held, it did not provide education about gaseous hazardous materials, including chlorine gas, which could be produced as byproducts of manufacturing processes.

Secondly, instructions about safe evacuation routes were not sufficiently provided. The epicenter of the chlorine gas production was near the main staircase on the first floor. Some workers who attempted evacuation through the main staircase without knowing these facts were confined in the passage or evacuated to the rooftop and thus were exposed to high doses of chlorine gas. For this reason, 6 subjects in the high-exposure group were exposed to higher doses even though they attempted evacuation immediately after recognizing the gas leakage. The exposure time would have been within 5 min if they had evacuated through the opposite emergency exit instead of the main staircase. The cause of this also included the problem that evacuation announcement was not effectively used. Although the person who was in charge of ordering evacuation at the scene explained that it would be faster to directly order the evacuation instead of looking for the broadcasting facility, as it was a small building, eventually the safety of the workers moving according to the evacuation order was not guaranteed and appropriate guidelines were not provided to all the workers.

It was postulated that chlorine gas would take about 90 min to diffuse. The firefighter team, which was the first to be mobilized after the accidental leakage was reported, did not have a device to measure chlorine gas and thus could not measure the accurate concentration. However, it is assumed that they were exposed to about 3 to 10 ppm chlorine gas concentrations based on the reports and symptoms of the victims [5]. Although the leakage was accidental, only few cases were severe, as the chlorine gas concentration was relatively lower than in other accidental leakages. However, based on the result of the analysis of the evacuation behaviors of the workers, we postulated that the leakage would have led to a major accident if the amount of chlorine gas emission was large. Although the leakage of sodium chlorate can be considered as a direct cause, the accident led to casualties due to the potential drawbacks such as the pipe arrangement without by-product consideration, lack of a ventilation system for gaseous hazardous materials, lack of education for workers, and lack of protective gears [18, 19].

The limitations of this study are as follows: First, although the exposure time and behavioral characteristics were accurately reenacted through a self-administered survey and individual interview with occupational medicine specialists, this was limited in that the victims’ memories were unreliable. More accurate results could have been obtained if the evaluation had been performed immediately after the incident. Second, although the study subjects were healthy adults without history of respiratory diseases, the duration of subjective acute respiratory symptoms did not exactly reflect the dose, as the symptoms of chlorine gas exposure were nonspecific and varied between the individuals. Third, this study estimated the exposure time from the beginning of chlorine gas exposure to the completion of evacuation to the safety zone. This can be different from the actual exposure time. However, the discrepancy was considered minimal considering the characteristics of chlorine gas, which diffuses rapidly, and the factory size and evacuation route.

## Conclusions

In accidental chlorine gas leakage, the duration of the subjective acute respiratory symptoms significantly differed between the low- and high-exposure groups. Based on our investigation of the reasons for the difference in evacuation behaviors of 27 highly exposed workers, it appears that evacuation was most commonly delayed due to misjudgment of toxicity after recognizing the leaked gas. Additionally, in case where evacuation was delayed by helping other evacuators, people were highly exposed because the evacuation route was not appropriate even though they attempted immediate evacuation. In order to prevent such a disaster due to accidental leakage of hazardous agent, workers should be educated about not only the agents that are directly dealt during the manufacturing process but also the hazardous agents that can be produced as by-product
